# Single and Combined Effects of Chlorpyrifos and Glyphosate on the Brain of Common Carp: Based on Biochemical and Molecular Perspective

**DOI:** 10.3390/ijms241612934

**Published:** 2023-08-18

**Authors:** Dongfang Zhang, Weikai Ding, Wei Liu, Liuying Li, Gongming Zhu, Junguo Ma

**Affiliations:** 1 Henan International Joint Laboratory of Aquatic Toxicology and Health Protection, College of Life Science, Henan Normal University, Xinxiang 453007, China; 2 State Key Laboratory of Antiviral Drugs, Henan Normal University, Xinxiang 453007, China; 3 Pingyuan Laboratory, Xinxiang 453007, China

**Keywords:** *Cyprinus carpio*, chlorpyrifos, glyphosate, combined exposure, neurotoxicity

## Abstract

Chlorpyrifos (CPF) and glyphosate (GLY) are the most widely used organophosphate insecticide and herbicide worldwide, respectively; co-occurrence of CPF and GLY in aquatic environments occurs where they inevitably have potential hazards to fish. However, the potential mechanisms of CPF and GLY to induce toxicity have not been fully explored. To identify the adverse impacts of CPF and GLY on fish, either alone or in combination (MIX), CPF (25 μg/L) and GLY (3.5 mg/L) were set up according to an environmentally relevant concentration to expose to common carp for 21 days. After exposure, CPF and GLY decreased the activities of acetylcholinesterase and Na^+^/K^+^-ATPase, altered monoamine oxidase levels, decreased antioxidant enzyme activities (superoxide dismutase, catalase, glutathione S-transferase and glutamic reductase), and induced the accumulation of malondialdehyde in the carp brain. The parameters in the MIX groups had a greater impact compared to that in the CPF or GLY group, suggesting that both single and combined exposure could affect neurological signaling systems and cause oxidative stress and lipid peroxidation damage in carp brains, and that MIX exposure increases the impact of each pollutant. RNA-seq results showed that single or combined exposure to CPF and GLY induced global transcriptomic changes in fish brains, and the number of differentially expressed genes in MIX-treated carp brains were globally increased compared to either the CPF or GLY groups, suggesting that the effects of co-exposure were greater than single exposure. Further analysis results revealed that the global transcriptomic changes participated in oxidative stress, immune dysfunction, and apoptosis of fish brains, and identified that the P13k-Akt signaling pathway participates in both single and combined exposure of CPF- and GLY-induced toxicity. Taken together, our results demonstrated that the interaction of CPF and GLY might be synergic and provided novel insights into the molecular mechanisms of fish brains coping with CPF and GLY.

## 1. Introduction

The residues of pesticides in the aquatic environment have been widely considered due to their potential threats to wild organisms and even human health [1,2]. Chlorpyrifos (CPF) is a class of organophosphorus pesticides (OPs) used for pest control worldwide by farmers and city inhabitants [3,4] which has been frequently detected in the natural environment. For example, CPF and its degradation products (13.4 ng/L) were found in lake water in Vietnam [5] and up to 5.49 µg/L CPF was found in Buenaventura ditch water in Mexico [6]. What is more, up to 0.20 μg/g CPF residue was detected in a fish sample from the Tono reservoir, Ghana [7] and 0.053 μg/g CPF was found in a fish sample in the Chilika Lake, India [8]. CPF is reported to be highly toxic to aquatic animals, especially fish, since this insecticide can be absorbed quickly through the gills, skin, and digestive system by fish [9]. The recognized toxic mechanism of CPF is that it can inhibit the activity of acetylcholinesterase (AChE), resulting in severe adverse impacts on non-target organisms, e.g., nephrotoxicity, immunotoxicity, genotoxicity, reproductive, and developmental toxicity [10,11,12].

Glyphosate (GLY) is one of the most extensively used OPs in the agricultural field worldwide that acts in plant’s 5-enol-pyruvilshikimato-3-phosphatosintase enzyme, which is absent in animals [13]. Therefore, GLY is thought to be non-toxic or have a relatively low toxicity for animals and humans. However, recent studies have demonstrated that GLY is moderately toxic to aquatic organisms [14]. GLY exerts a significant toxic affect on the nervous system of fish [15]. The alteration of carbonic anhydrase (CA), AChE, and Na^+^/K^+^-ATPase levels, and apoptosis have been described in fish following GLY exposure [16,17]. In recent years, GLY is commonly detected in natural waters due to its widespread use, e.g., almost all detected water samples (99.9% each) contained GLY, aminomethyl phosphonic acid (which is the metabolites of GLY), or both compounds [18]. GLY was also found in 1.01% of 694 groundwater samples (in maximum concentrations of up to 2.09 μg/L) and detected in 14.3% of 196 surface water samples (with maximum contents of 32.49 μg/L) in agricultural basins in ten Chinese provinces [19]. The average concentrations of GLY commonly detected in fresh surface waters range from 0.13 to 36.71 μg/L worldwide [20]. However, levels as high as 15.21 mg/L GLY were detected in water from Lake Taihu in China [21], 5.2 mg/L in large streams and groundwater in the European Union [22], and 1 mg/L in water from Békés County in Hungary [23]. Consequently, co-occurrence of GLY and CPF in various aquatic habitats could happen where it may unavoidably affect aquatic creatures, such as fish; the negative impacts and knowledge of the probable harmful processes of CPF and GLY to fish are still relatively rare.

Common carp (*Cyprinus carpio*) were selected as a candidate species to study the potential toxic effects of CPF and GLY in aquatic environments because common carp are a commonly used model for toxicological studies [24,25] and they are easily captured and important for human consumption. The brain is the central organ controlling the physiology of the animal and plays a crucial role in maintaining normal physiological processes, highlighting its importance in environmental risk assessments [26]. The enzyme and protein levels of the brain are frequently used as sensitive indicators of toxicity in aquatic environments and are suitable candidates to appraise the consequences of pesticides on fish [27,28]. In the present study, common carp brains were selected to investigate biochemical and transcriptomic alterations to evaluate the toxic effects and mechanisms of low concentrations of CPF and GLY alone or in combination on fish. The study refreshes the cognition related to the mechanisms of single or combined exposure of CPF- and GLY-induced brain injuries in fish and will provide essential data for ecological risk assessment and the rational application of CPF and GLY.

## 2. Results

### 2.1. Contents of CPF and GLY

The contents of CPF and GLY in the fish brain are displayed in Table S1. The average concentration of CPF in the brain was 2.59 and 4.07 ng/g in the CPF groups, and 1.28 and 2.17 ng/g in the MIX groups after 14- and 21-day exposure, respectively. After 14- and 21-day exposure, the average concentration of GLY in the fish brain was 113.61 and 117.82 ng/g in the GLY groups, respectively, and the average content of GLY in the MIX groups was 80.47 and 104.78 ng/g, respectively.

### 2.2. AChE Activities in the Fish Brain

**Figure 1:**
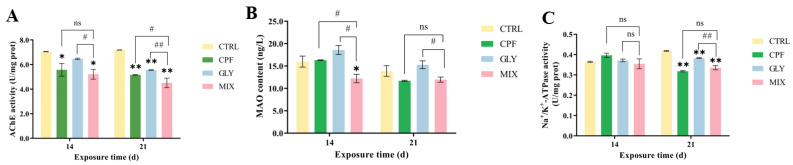
Effects of CPF, GLY, and MIX exposure on the AChE, MAO, and Na^+^/K^+^-ATPase levels in the carp brain. (**A**) AChE activities. (**B**) MAO. (**C**) Na^+^/K^+^-ATPase. * *p* < 0.05 and ** *p* < 0.01 represent significant differences between the CPF, GLY, MIX, and CTRL groups. # *p* < 0.05, and ## *p* < 0.01 represent the levels of difference between the MIX and CPF or GLY groups, respectively, “ns” represents not significant.

The AChE activities in the carp brain were significantly decreased in the CPF-, GLY-, and MIX-treated groups compared to the control (CTRL) groups (except for the GLY-treatment groups at 14 days) (Figure 1A). Moreover, at 21 days, the MIX groups had more noticeable AChE changes than the solo groups.

### 2.3. Monoamine Oxidase (MAO)

The MAO levels in the MIX-treated fish brain were markedly inhibited at 14 days but not markedly altered in the CPF and GLY groups compared to the CTRL groups; they were not noticeably altered at 21 days in the CPF, GLY, and MIX groups (Figure 1B).

### 2.4. Na^+^/K^+^-ATPase

The activities of Na^+^/K^+^-ATPase were not substantially altered following a 14-day exposure of CPF, GLY, and MIX, but they were significantly suppressed after 21 days of CPF, GLY, and MIX exposure, compared to the CTRL (Figure 1C). Meanwhile, the Na^+^/K^+^-ATPase activities in the MIX groups were markedly lower than the activities of Na^+^/K^+^-ATPase in the GLY groups.

### 2.5. Oxidative Stress-Related Indicators in the Brain

**Figure 2:**
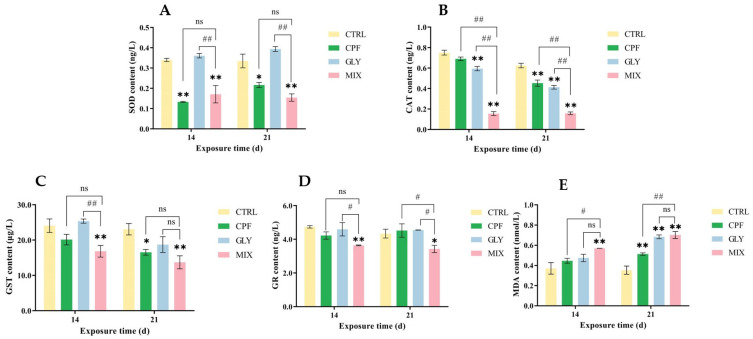
Effects of CPF and GLY single or co-exposure on the levels of oxidative stress-related indicators in the carp brain. (**A**) SOD levels. (**B**) CAT. (**C**) GST. (**D**) GR. (**E**) MDA. Expressions of the difference in levels between the CPF, GLY, MIX groups and CTRL groups are * *p* < 0.05 and ** *p* < 0.01. # *p* < 0.05 and ## *p* < 0.01 represent the levels of difference between the MIX and CPF or GLY groups, “ns” represents not significant.

The findings of the assay for oxidative stress markers revealed a substantial difference between the CPF, GLY, MIX groups and the CTRL groups (Figure 2). As shown in Figure 2, when compared to the CTRL groups, the superoxide dismutase (SOD) levels in the CPF and MIX groups were significantly inhibited; there was no significant change in the GLY groups (Figure 2A). The catalase (CAT) levels were generally inhibited in the CPF, GLY, and MIX groups, while the alterations were more pronounced in the MIX groups than in the separate groups (Figure 2B). In addition, the levels of glutathione S-transferase (GST) and glutamic reductase (GR) in the fish brain were significantly decreased in the MIX groups but not markedly changed in the CPF and GLY groups (except for GST in the CPF groups at 21 days) (Figure 2C,D). Meanwhile, the contents of malondialdehyde (MDA) were generally enhanced in the CPF, GLY, and MIX groups, and the alterations of MDA were more pronounced in the MIX groups than in the single groups (Figure 2E).

### 2.6. RNA-seq Data

Twelve cDNA libraries were constructed from the total RNA extracted from the brain of the CTRL, CPF, GLY, and MIX groups and 45,872,406 to 57,312,482 raw reads were obtained from RNA sequencing (Table S2). A total of 44,589,422 to 55,700,310 clean reads were produced after quality control. The carp reference genome was then mapped using high-quality reads: a total of 47,391,426 to 52,828,442 reads (94.79–94.96%) of the CTRL groups, 43,876,286 to 52,302,296 reads (94.76–94.96%) of the CPF groups, 43,460,902 to 51,603,790 reads (94.61–94.95%) of the GLY groups, and 42,320,019 to 43,966,057 reads (94.91–95.05%) of the MIX groups were obtained.

### 2.7. DEGs and qPCR Validation

**Figure 3:**
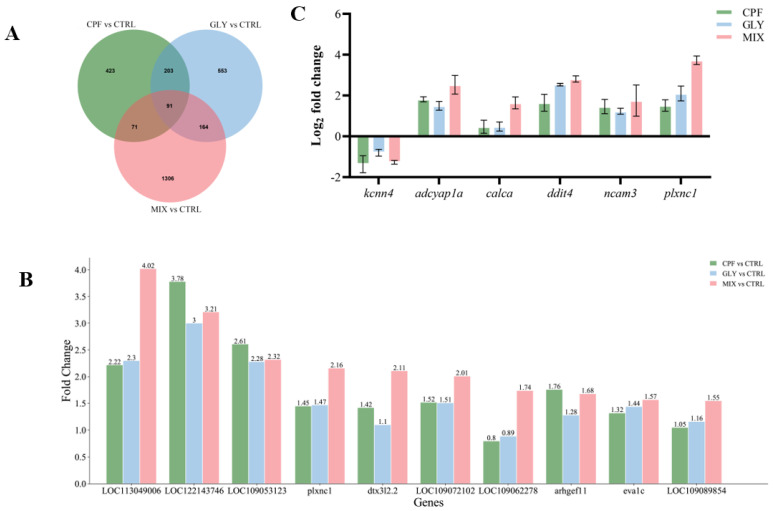
Transcription changes after CPF, GLY, and MIX exposure. (**A**) Venn diagram illustrating DEGs overlap for the three exposed groups (|log2(fold change)| > 1.5 and *p*-value < 0.05). (**B**) The top 10 up-regulated fold changes in DEGs in the CPF, GLY, and MIX groups. (**C**) Verification of the RNA-seq results using qPCR.

On the basis of the screening standard (|log_2_(fold change)| > 1.5 and *p* < 0.05), 788 (upregulated 484 genes and downregulated 304 genes), 1011 (667 genes upregulated and 344 genes downregulated), and 1632 (upregulated 1317 genes and downregulated 315 genes) DEGs were found in the fish brain after CPF, GLY, and MIX exposure, respectively (Figure S1A–C). A total of 91 genes were altered in all three exposure groups; each treatment group had a majority of unique transcripts (Figure 3A) which may indicate some similarity in the modes of action of CPF, GLY, and MIX. A smaller subset of the top 10 upregulated DEGs was present in all three exposure groups (Figure 3B). The MIX groups had a greater global impact on these genes, which is consistent with the results of the biochemical study above. We separately analyzed the DEGs of MIX vs. CPF and MIX vs. GLY to identify the difference between the combined and single exposure of CPF and GLY. When compared to CPF, the MIX treatment upregulated 994 genes and downregulated 422 genes (Figure S1D); when compared to GLY, the MIX treatment upregulated 830 genes and downregulated 444 genes (Figure S1E), in which there were 489 DEGs that overlapped in the MIX vs. CPF and MIX vs. GLY analysis as shown in Figure S1F.

Six of these overlapping DEGs (*kcn4*, *adcyap1a*, *calca*, *ddit4*, *nacam3*, and *plxnc1*) were randomly selected and verified by qPCR. The expression levels of all 6 genes as measured by qPCR were consistent with the RNA-Seq data (Figure 3C) and Pearson’s bivariate correlation analysis revealed a high correlation between qPCR and RNA-seq data (Figure S1G).

### 2.8. GO Classification and KEGG Enrichment Analysis of DEGs

**Figure 4:**
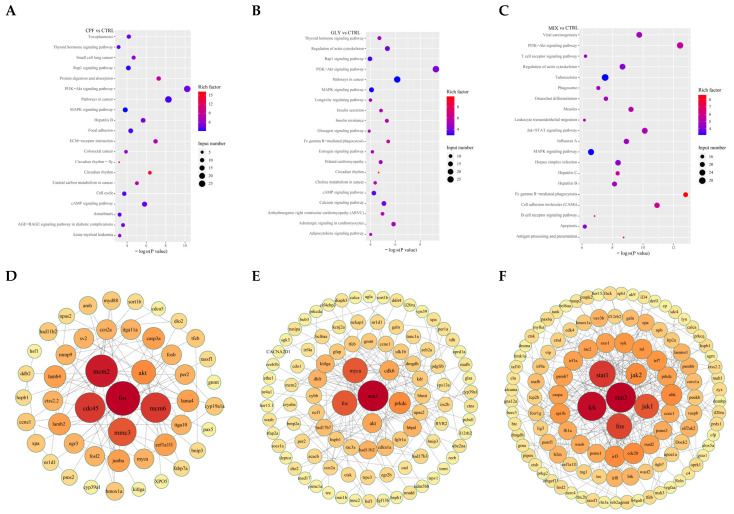
KEGG enrichment analysis of DEGs in CPF, GLY, and MIX treatment groups. (**A**–**C**) KEGG enrichment analysis of DEGs in the CPF, GLY, and MIX groups. Significantly enriched KEGG pathways are represented on the *y*-axis, while the richness of DEGs is represented on the *x*-axis. The size and color of the dispersed dots correspond to the number of genes and the Q-value, respectively. (**D**–**F**) The hub genes in the CPF, GLY, and MIX groups were classified by PPI network research. The size and color of the dots correspond to the degree of genes.

The GO terms “growth”, “nervous system development” and “extracellular matrix structural constituent” were considerably enriched in the genes that showed differential expression after CPF treatment (Figure S2A). The DEGs in CPF exposure groups were connected to the “P13K-Akt signaling pathway”, “Focal adhesion”, “Cell cycle”, and “cAMP signaling pathway”, per the categorization and enrichment analysis of KEGG pathways (Figure 4A). Protein–protein interaction (PPI) network research revealed that the top hub genes (Figure 4D) in these pathways were *cdc45*, *mcm6*, *mcm3*, *mcm2*, *fos*, and *akt* (Table S3), which are crucial for promoting the survival of neurons; the expression of these genes was markedly reduced by CPF.

DEGs in GLY treatment groups were markedly enriched in the GO terms “negative regulation of response to stimulus”, “negative regulation of signal transduction”, and “negative regulation of cell communication” (Figure S2B), and are associated with the KEGG pathways “P13k-Akt signaling pathway”, “Glucagon signaling pathway”, “Calcium signaling pathway” and “Fc gamma R-mediated phagocytosis” (Figure 4B). PPI network research revealed that the DEGs in these pathways included *stat3*, *myc*, *fos*, *cdk6*, *prkdc*, and *akt*, in which *stst3* and *prkdc* were significantly upregulated by GLY (Figure 4E, Table S3).

In MIX groups, EDGs were markedly enriched in the GO terms “immune response”, “purine ribonucleoside triphosphate binding”, and “intracellular signal transduction” (Figure S2C), and connected to the “P13K-Akt signaling pathway”, “Fc gamma R—mediated phagocytosis”, “Apoptosis”, and “Cell adhesion molecules” KEGG pathways (Figure 4C). PPI network research revealed that the hub genes in these pathways were *lck*, *stat1*, *stat3*, *jak1*, *jak2*, and *fos* (Figure 4F, Table S3). The expression of these genes was markedly increased by MIX and most of the top hub genes (*stat1*, *stat3*, *ja1*, and *jak2*) were enriched in the necroptosis.

## 3. Discussion

CPF and GLY are frequently detected in natural water and might endanger the health of aquatic creatures. However, data on the potential toxic mechanisms of CPF and GLY on fish are relatively scarce. In the present study, we mainly reported the biochemical effects of single and combined exposure of CPF and GLY on common carp brains. Transcriptomic analysis has also been used to reveal previously unknown information about the toxic mechanisms of these substances on fish. Our results showed that both CPF and GLY are detected in carp brain tissue (Table S1). This evidence, in conjunction with previous studies on the CPF effects on an in vitro model of the human blood–brain barrier (BBB) and GLY exposure on mice, suggest that CPF and GLY can cross the BBB and enrich carp brain tissue [29,30]. Given the detection of CPF/GLY in the brain, the next step was to determine whether CPF and GLY affect the central nervous system (CNS),causing neurotoxicity in carp.

AChE and MAO play a crucial role in neurotransmitter release, synaptic plasticity, and the regulation of neuronal electrical activity in CNS [31]. AChE is an enzyme that hydrolyzes the neurotransmitter acetylcholine (ACh). ACh builds up at synapses and neuromuscular junctions as a result of AChE inhibition, which causes cholinergic fibers to be continuously stimulated throughout the nervous system until they are overstimulated and die [32,33]. In the present study, the activities of AChE in the fish brain were generally inhibited in the CPF, GLY, and MIX groups as compared to the CTRL groups (Figure 1A), which may lead to an accumulation of the ACh in the nervous system and then lead to impairment of the CNS and induce neurotoxicity [34,35]. AChE activities in the carp brain treated with CPF were generally lower than AChE activities in the fish brain exposed to GLY, and the inhibition of AChE was more pronounced in the MIX groups than in the single groups (Figure 1A), which may be due to the fact that CPF is an alkyl phosphate compound that can directly inhibit AChE function via the phosphorylation of serine residue at the catalytic domain of the enzyme [36], while GLY lacks the specific chemical groups that contribute to AChE inhibition; the effect of GLY on AChE may be attributed to the indirect effect of glycine present in its chemical structure [37], and the CPF and GLY may elicit synergistic effects on the inhibition of AChE.

MAO is an essential enzyme to catalyze the oxidations of monoamine neurotransmitters in the brain that are crucial for the usual continuance of the nervous system [38], which is also considered as a sensitive marker of neural dysfunction [39]. Previous research suggested that various xenobiotic compounds may inhibit MAO by reacting with the thiol (-SH) groups in the enzyme active sites [40]. In this study, the MAO level was not markedly altered in the CPF and GLY groups but was significantly inhibited in the MIX-treated fish brain at 14 days, compared to the CTRL groups (Figure 1B). The inhibition of MAO levels following MIX exposure may occur due to the alteration of available free-SH groups in the enzyme [41], causing neural dysfunction. With prolonged exposure, the levels of MAO were not noticeably altered at 21 days in the CPF, GLY, and MIX groups, which might be due to an adaptive regulation of the fish.

Na^+^/K^+^-ATPase, a critical enzyme for CNS functioning, is responsible for the active transport of Na^+^ and K^+^ in the nervous system and is essential for neuronal activity at nerve endings [42]. Changes in the activities of Na^+^/K^+^-ATPase in cerebral synaptosomes may compromise synaptic activities [43]; thus, it is frequently considered to be a sensitive indicator of toxicity [17]. In the present study, the activities of Na^+^/K^+^-ATPase were not noticeably changed following a 14-day exposure of CPF, GLY, and MIX, which might be due to an adaptive regulation of the fish; they were significantly suppressed at 21 days, compared to the CTRL (Figure 1C). This might disturb the Na^+^/K^+^ pump, resulting in limitation of the Na^+^/K^+^-ATPase synthesizing capability and impacting neuronal and excitatory transmission [43], suggesting that CPF, GLY, and MIX exposure may cause neurotoxicity in carp.

Previous studies have demonstrated that the attenuation of Na^+^/K^+^-ATPase activity may be due to the direct interaction of xenobiotic compounds with the enzyme [44] and also due to the raised lipid peroxidation which, in turn, impairs the structure of Na^+^/K^+^-ATPase [45,46,47]. In previous studies, single CPF or GLY treatment could initiate or exacerbate the generation of reactive oxygen species (ROS) and oxidative stress and then causes oxidative damage in fish [48,49]. SOD, CAT, GST, and GR are considered as the most efficient antioxidants that play an important role in maintaining ROS balance and protecting organisms from oxidative injury [17]; they are frequently used as biomarkers due to their sensitive response in order to evaluate the toxicity under xenobiotic stress. In the current study, the levels of SOD, CAT, GST, and GR were generally inhibited after CPF, GLY, and MIX exposure, and the levels of those indicators were significantly lower in the MIX groups, compared to the CTRL (Figure 2A–D). The decrease in the total antioxidant level might be due to compensatory mechanisms for the impact of ROS on the antioxidant system, which may cause oxidative stress and even lipid peroxidation in fish brains. In terms of the CAT activity, the combined effect seems to be prominent, which may be due to the overproduction of ROS caused by the combined CPF and GLY, leading to increased H_2_O_2_ in the brain cells which consumes a lot of CAT. It may also be due to the direct interaction of CPF and GLY with the CAT enzyme, although further experiments are needed to reveal this. MDA is the final product of lipid peroxidation and its increased level indicates the enhanced production of free radicals and injury to cell membranes [50]. The increased MDA level in the fish brain in this study indicates lipid peroxidation or even oxidative damage in fish exposed to CPF, GLY, and MIX (Figure 2E). The co-exposure of CPF and GLY caused a substantial rise in MDA levels, demonstrating further that the MIX amplifies lipid peroxidation effects, suggesting that both single and combined exposure could cause oxidative stress and lipid peroxidation damage in the carp brain, and that the MIX increases the impact of each pollutant.

To further reveal the toxic mechanisms of CPF, GLY, and MIX on the fish brain, the transcriptional profile was examined by RNA-seq. In the present study, 788 (484 upregulated genes and 304 downregulated), 1011 (667 upregulated and 344 downregulated), and 1632 (1317 upregulated and 315 downregulated) DEGs were found in the fish brain after CPF, GLY, and MIX exposure, respectively (Figure S1A–C). The qPCR results of the randomly selected DEGs were consistent with the RNA-seq results (Figure 3C), indicating the accuracy and reliability of the RNA-seq results obtained in this study. The single or combined exposure of CPF and GLY caused remarkable alterations on the transcriptome of brain. In this study, the RNA-seq results revealed that the number of DEGs and the amplitude of the changes in the gene expression in the MIX groups globally increased when compared to either CPF or GLY solely (Figure 3B), suggesting that the effects of co-exposure were greater than the single exposure.

Under CPF exposure, transcriptomic analysis results indicated that the PI3K-Akt signaling pathway, cAMP signaling pathway, focal adhesion, and cell cycle were significantly enriched (Figure 4A). Earlier investigations have demonstrated that the PI3K/AKT/JNK pathway participates in the toxicity of CPF [51,52]. Furthermore, CPF could induce oxidative stress, apoptosis, and immune dysfunction in fish [53], which is also consistent with what we discovered in the present study. Meanwhile, in the CPF-treated fish, KEGG pathways with the hub genes *cdc45*, *mcm2*, *mcm3*, *mcm6*, and *fos* were included in the cell cycle, and the membrane translocation/activation of *akt* was essential in neuronal survival [54]. *Fos*, which encodes the transcription factor *c-fos*, can operate as a “third messenger” to regulate the expression of target genes, which has been linked to the effect of neuronal and glial cell proliferation, growth, inflammation, injury, and other circumstances [55], suggesting that CPF exposure might affect cell cycles in fish brains and that the neurotoxic mechanisms of CPF are intricate.

In our study, GLY exposure significantly affects the immune system and the endocrine system, such as Fc gamma R-mediated phagocytosis and the glucagon signaling pathway (Figure S2B), which has also been found in other fish after GLY exposure [56,57]. In addition, the calcium signaling pathway and the P13k-Akt signaling pathway have been effected by GLY (Figure 4B). It was reported that PI3K-Akt plays an important role in mediating inflammation, oxidative stress, BBB dysfunction, and apoptosis of neurotoxicity in carp [58], and the calcium signaling pathway also participates in the toxicity mechanism of OPs [59], suggesting that the P13k-Akt and calcium signaling pathway might be involved in the regulation of brain injury induced by GLY exposure. The hub genes in the significant KEGG pathways after GLY exposure were similar to CPF, compared with the CTRL, which may be related to the fact that they are both OPs and have similar mechanisms of action.

Compared to the CTRL groups, “immune response”, “purine ribonucleoside triphosphate binding”, “intracellular signal transduction”, “P13K-Akt signaling pathway”, “Fc gamma R—mediated phagocytosis”, “Apoptosis” and “Cell adhesion molecules” were significantly enriched in the MIX groups (Figure 4C). In addition, according to the results of the qPCR combined with the transcriptome analysis, it was found that the MIX exposure amplified the single toxic effect of CPF or GLY, and most of the top hub genes (*stat1*, *stat3*, *jak1*, and *jak2*) were all enriched in the necroptosis. In the etiology of various neurodegenerative illnesses, including multiple sclerosis, amyotrophic lateral sclerosis, Parkinson disease’s, and Alzheimer’s disease, necroptosis promotes additional cell death and neuroinflammation [60]. Necrosis has the potential to accelerate the degradation of axons, which are important targets for neuroprotection [61]. In this study, the DEGs in the MIX treatment groups were significantly enriched in the pathways of cell death, suggesting that MIX exposure may disrupt fish cranial nerves primarily by affecting neuronal cell death in carp.

Overall, the KEGG analysis indicated that multiple pathways were changed at the transcriptional level in both single and combined exposure of CPF and GLY, and the P13K-Akt signaling pathway was enriched in both single and combined exposure of CPF and GLY, suggesting that the impacts of CPF and/or GLY exposure on the fish were intricated and that the P13K-Akt signaling pathway may play an important role in CPF- and/or GLY-induced neurotoxicity, although in-depth functional research is needed to reveal specific signaling pathways and the cross-talk interactions of pathways.

## 4. Materials and Methods

### 4.1. Chemicals

Chlorpyrifos (CPF, #C109843, purity 99%, Aladdin Bio-Chem Technology Co., Ltd., Shanghai, China) and glyphosate (GLY, #BCBZ6585, purity ≥ 98%, Sigma-Aldrich Co., St Louis, MO, USA) were prepared from standard grade stocks in ultrapure water.

### 4.2. Fish and Experimental Design

Juvenile common carp were purchased from the fish farm in Yellow River Basin, Xinxiang, Henan Province, China, and grown in 12 laboratory tanks (250 L-60 in each tank, pH 7.22 ± 0.08, temperature 25 ± 1 °C) at random. Before the lab started, the fish were fed with a commercial diet at 3% the weight of the fish twice a day, with regular feeding at 8:00 am and 3:00 pm every day during the adaptation period. After acclimation, 240 carps (3.87 ± 0.65 cm, 6.07 ± 0.75 g) were randomly split into 4 groups, including 25 μg/L CPF, 3.5 mg/L GLY, MIX (25 μg/L CPF + 3.5 mg/L GLY), and CTRL groups. Ecologically relevant concentrations described in the introduction were used to establish the exposure concentrations of CPF and GLY (also refer to the previous literature [14]). The experiments were repeated thrice for each treatment. Samples of the exposure solutions were collected at the beginning and the end of the experiments, and the content of the substances was measured by high-performance liquid chromatography in the case of CPF and GLY, as previously described [62,63]. The actual concentrations of CPF and GLY were: CPF group (24.15 ± 0.48 μg/L), GLY group (3.42 ± 0.07 mg/L), and MIX group (CPF 24.0 ± 0.69 μg/L, GLY 3.35 ± 0.12 mg/L). To maintain a consistent toxicant exposure concentration in the water from the start of the experiment until its conclusion, four-fifths of the exposure solution was replaced every day.

Carps were anesthetized after being exposed for 14 and 21 days, and after weighing the brain tissue, it was promptly preserved at −80 °C for later analysis. All treatments were approved by the Ethics Committee of Henan Normal University.

### 4.3. Determination of CPF and GLY Contents in Carp Brain

The ELISA kits (#m1855162, #m1621570) were used to identify the contents of CPF and GLY in the fish brain, according to the manufacturer’s instructions (Shanghai Enzyme Biotechnology Co., Ltd., Shanghai, China).

### 4.4. Biochemical Assays

The carp brain tissues were homogenized in 0.9% ice-cold NaCl solution (a ratio of 1:9 *w*/*v*) and centrifuged at 2500× *g* for 10 min at 4 °C and then the supernatant was collected for biochemical analysis. The activities of AChE (#A024-1-1) and Na^+^/K^+^-ATPase (#A070-2) in brain tissue homogenate were measured by physiological biochemical kits (Nanjing Jiancheng Bioengineering Institute, Nanjing, China).

The related ELISA kits were used to detect the levels of superoxide dismutase (SOD) (#E-42689), catalase (CAT) (#E-42495), glutathione S-transferase (GST) (#E-53709), glutamic reductase (GR) (#E-53710), monoamine oxidase (MAO) (#E-53776), and malondialdehyde (MDA) (#E-42461), and the operation procedures were performed according to the kit’s instructions (Beijing Andy Huatai Technology Co., Ltd., Beijing, China).

### 4.5. Transcriptomic Analysis

After 21-day exposure, the carp brains were collected for the transcriptome study. Five carp brains were combined for each sample to create a sufficient mass for total RNA extraction (Invitrogen, Carlsbad, CA, USA, cat. NO 15596026). Quality control of the RNA samples, RNA-seq experiments, and high throughput sequencing and data analysis were conducted by Seqhealth Technology Co., Ltd. (Wuhan, China). Using the KCTM Stranded mRNA Library Prep Kit for Illumina^®^ (Catalog NO. DR08402, Wuhan Seqhealth Co., Ltd., Wuhan, China) following the manufacturer’s instructions, stranded RNA sequencing libraries were prepared using 2 μg of total RNA. A DNBSEQ-T7 sequencer (MGI Tech Co., Ltd., Shenzhen, China) with the PE150 model was used to enrich, quantify, and finally sequence PCR products with a bandwidth of 200–500 bps. After that, the library was sequenced. Clean data with a high quality were mapped to the reference genome of *Cyprinus carpio* using STRA software (version 2.5.3a) with default parameters. A *p*-value cut-off of 0.05 and a fold-change cut-off of 1.5 were used to judge differentially expressed genes (DEGs). Gene ontology (GO) analysis was implemented by KOBAS software (version:2.2.2) with a *p*-value cutoff of 0.05 to judge statistically significant enrichment. A Kyoto encyclopedia of genes and genomes (KEGG) enrichment analysis also with a *p*-value cutoff of 0.05 was used to judge statistically significant enrichment and was implemented by KOBAS software (version:2.2.2) and by a free online platform (http://www.bioinformatics.com.cn accessed on 1 November 2022). The raw data generated from RNA-seq are available in the NCBI Sequence Read Archive (SRA). The RNA-sequencing data can be found at https://www.ncbi.nlm.nih.gov/sra/PRJNA1004878 accessed on 13 August 2023 with accession number PRJNA1004878.

### 4.6. Real-Time Quantitative PCR (qPCR)

Fish brains for each treatment were collected to extract RNA using a RNAiso Kit (#R0026, Beyotime Biotech Inc., Shanghai, China). For each sample, 0.5–2 µg of high-quality total RNA was subjected to reverse transcription using the Vazyme cDNA Synthesis Kit (#R223-01, Nanjing, China). We used 96-well plates, each well with a total volume of 10 µL, including 1 µL cDNA as template, 0.4 µL F primer, 0.4 uL R primer, 3.2 µL nuclease-free water, and 5 µL master mix (#Q511, Vazyme biotech Co., Ltd., Nanjing, China). The primer sequences utilized for the six genes (*kcn4*, *adcyap1a*, *calca*, *ddit4*, *nacam3*, and *plxnc1*) are presented in the supplemental table (Table S4). Primers were designed by Primer-BLAST (https://www.ncbi.nlm.nih.gov/tools/primer-blast/ accessed on 1 October 2022) and purchased from Sangon Biotech (Shanghai, China). The qPCR experiments were performed following the MIQE guidelines [64], and the transcript levels for target mRNA were computed by the 2^−ΔΔCT^ method [65].

### 4.7. Statistical Analysis

All statistical analyses were performed using IBM SPSS Statistics 20.0 (IBM, Armonk, NY, USA) software. A Shapiro–Wilk test and Levene’s test were used to determine the normal distribution and homogeneity of variance, respectively. All data were statistically examined using one-way analysis of variance (ANOVA). The standard errors of the mean (SEM) are shown as deviation bars. Asterisks (*) represent significant differences for GLY, CPF, and MIX groups compared with CTRL, hash symbol (#) represents significant differences between MIX and GLY or CPF.

## 5. Conclusions

Our results showed that single or combined exposure of CPF and GLY impacts the neurological signaling systems and causes oxidative stress and lipid peroxidation damage in carp brains. The PI3K-Akt signaling pathway might participate in this process and the combined exposure of CPF and GLY might amplify the single toxicity of CPF or GLY on carp brains. This study demonstrated that the interaction of CPF and GLY might be synergic, which is important for guiding us to evaluate the environmental risks of CPF and GLY.

## Data Availability

Data supporting the reported results are contained within the article.

## Supplementary Materials

The following supporting information can be downloaded at: https://www.mdpi.com/article/10.3390/ijms241612934/s1.
