# Supplementary material for: Single and Combined Effects of Chlorpyrifos and Glyphosate on the Brain of Common Carp: Based on Biochemical and Molecular Perspective
Source: Int J Mol Sci. 2023 Aug 18;24(16):12934. doi: 10.3390/ijms241612934 (PMC10455211; doi:10.3390/ijms241612934)
Supplement: Supplementary file 1 [file ijms-24-12934-s001.zip › ijms-2560223-supplementary.pdf]

1. Supplementary Tables

Table S1. CPF and GLY contents in the brain of common carp.

Exposure time (d)	Exposure	Contents of CPF and GLY in the brain(ng / g)	
		CPF	GLY
14	CTRL	-	-
	CPF	2.59±0.47	-
	GLY	-	113.61±9.18
	MIX	1.28±0.37	80.47±7.24
21	CTRL	-	-
	CPF	4.07±1.08	-
	GLY	-	177.82±29.25
	MIX	2.17±0.64	104.78±11.30

Table S2. Summary of transcriptome sequencing data

Sample	Raw Reads(bp)	Clean Data (bp)	Clean Reads %	Total Mapped	Uniquely Mapped	Q20 (%)	Q30 (%)	GC Content(%)
CTRL-1	51456144	49997446	97.17	47391426(94.79)	45882691(96.82)	100	98.65	48.5
CTRL-2	57312482	55700310	97.19	52828442(94.84)	51025436(96.59)	100	98.65	48.5
CTRL-3	56283346	54737816	97.25	51981699(94.96)	50182531(96.54)	100	98.7	48.5
CPF-1	49406378	48063430	97.28	45577031(94.83)	44011586(96.57)	100	98.75	48.5
CPF-2	56732800	55193288	97.29	52302296(94.76)	50607868(96.76)	100	98.7	48
CPF-3	47410510	46203714	97.45	43876286(94.96)	42415255(96.67)	100	98.8	48
GLY-1	55710116	54363108	97.58	51603790(94.92)	49828727(96.56)	100	98.9	48.5
GLY-2	52372360	51084614	97.54	48507065(94.95)	46895640(96.68)	100	98.85	48.5
GLY-3	47151062	45938928	97.43	43460902(94.61)	41996499(96.63)	100	98.8	48.5
MIX-1	45872406	44589422	97.2	42320019(94.91)	41018375(96.92)	100	98.65	48
MIX-2	47593102	46254978	97.19	43966057(95.05)	42564833(96.81)	100	98.7	48.5
MIX-3	45885388	48063430	97.35	42408938(94.94)	41155343(97.04)	100	98.75	48.5

TableS3. Hub genes in the CPF, GLY, and MIX groups compared to the CTRL group.

Group	Symbol	Name	KEGG_Orthology_Web
CPF	cdc45	cell division control protein 45	https://www.genome.jp/dbget-bin/www_bget?ko:K06628
	mcm2	DNA replication licensing factor MCM2	https://www.genome.jp/dbget-bin/www_bget?ko:K02540
	mcm3	DNA replication licensing factor MCM3	https://www.genome.jp/dbget-bin/www_bget?ko:K02541
	mcm6	DNA replication licensing factor MCM6	https://www.genome.jp/dbget-bin/www_bget?ko:K02542
	fos	proto-oncogene protein c-fos	https://www.genome.jp/dbget-bin/www_bget?ko:K04379
	akt	non-specific serine/threonine protein kinase	https://www.genome.jp/dbget-bin/www_bget?ko:K04456
GLY	stat3	signal transducer and activator of transcription 3	https://www.genome.jp/dbget-bin/www_bget?ko:K04692
	myc	Myc proto-oncogene protein	https://www.genome.jp/dbget-bin/www_bget?ko:K04377
	fos	proto-oncogene protein c-fos	https://www.genome.jp/dbget-bin/www_bget?ko:K04379
	cdk6	cyclin-dependent kinase 6	https://www.genome.jp/dbget-bin/www_bget?ko:K02091
	prkdc	DNA-dependent protein kinase catalytic subunit	https://www.genome.jp/dbget-bin/www_bget?ko:K06642
	akt	non-specific serine/threonine protein kinase	https://www.genome.jp/dbget-bin/www_bget?ko:K04456
MIX	lck	lymphocyte cell-specific protein tyrosine kinase	https://www.genome.jp/dbget-bin/www_bget?ko:K05856
	stat3	signal transducer and activator of transcription 3	https://www.genome.jp/dbget-bin/www_bget?ko:K04692
	stat1	signal transducer and activator of transcription 1	https://www.genome.jp/dbget-bin/www_bget?ko:K11220
	jak2	Janus kinase 2	https://www.genome.jp/dbget-bin/www_bget?ko:K04447
	jak1	Janus kinase 1	https://www.genome.jp/dbget-bin/www_bget?ko:K11217
	fos	proto-oncogene protein c-fos	https://www.genome.jp/dbget-bin/www_bget?ko:K04379

Table S4. Primer sequences used for qPCR analysis

Gene	Sequence (5'–3')		Product Size(bp)
kcnn4	F	GGGAGGACAAACTTGGGAGG	118
	R	AAGAACATGAGGGCCGACAG	
adcyap1a	F	GAAGGGAAATTGCGGTCGTG	165
	R	GGACGGATTTGAGGCGAGAT	
calca	F	CGAGGAAAACAGCCTGGGTA	114
	R	TTGGTGCGTGGATACGTCTG	
ddit4	F	CAGACGCGCACTTAGACTGA	149
	R	CGGAGGAGACCAAACGCTTA	
ncam3	F	CGGTGTGGTCGGGATTGTAA	193
	R	GAGGCCACTCAGCTTCATGT	
plxnc1	F	GTTCAATGAATCGGCGGCTC	156
	R	CCGCTTCGCTTCATGTTGTC	
β-actin	F	TGCTCTGTATGGCGCATTGA	137
	R	AGGGCAAAGTGGTAAACGCT	

2. Supplementary Figures

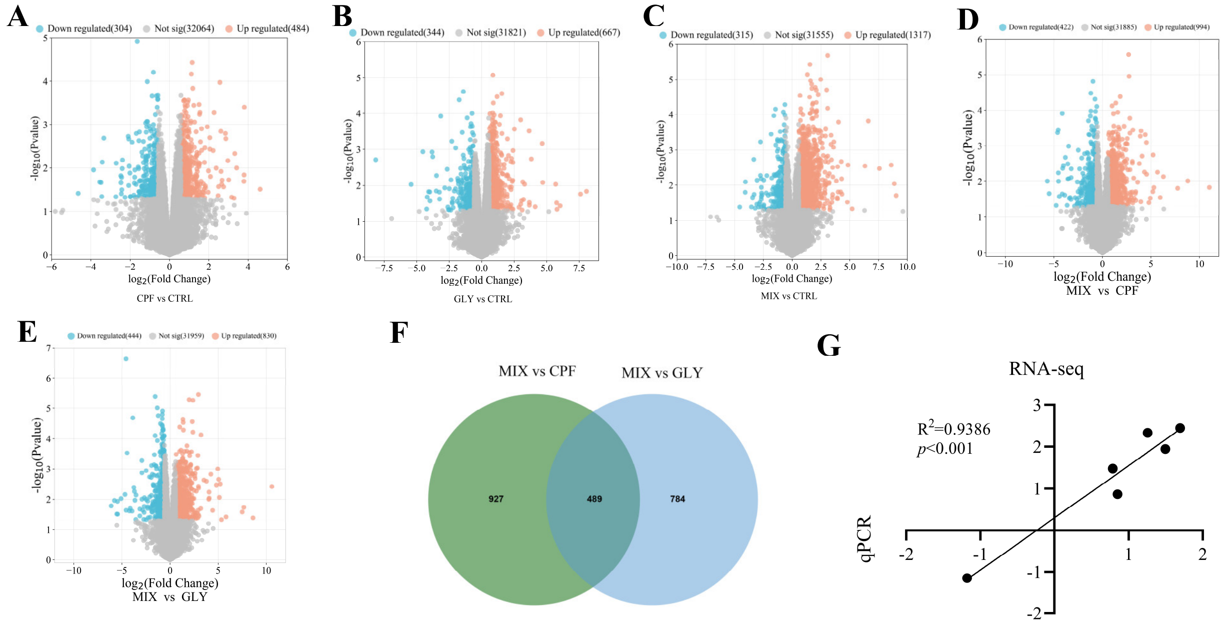

Figure S1. Transcription changes after CPF, GLY, and MIX exposure. (A) Volcano graphs of DEGs in the brain of carp following CPF exposure. (B) Volcano graphs of DEGs in the brain of carp following GLY exposure. (C) Volcano graphs of DEGs in the brain of carp following MIX exposure. Red dots denote genes that are considerably upregulated, blue dots denote genes that are significantly downregulated, and gray dots denote genes that are not significantly changed when compared to CTRL groups. (D) Volcano plot for MIX vs. CPF DEGs. (E) Volcano plot for MIX vs. GLY DEGs. (F) Venn diagram illustrating DEG overlap for the MIX compared with CPF groups and MIX compared with GLY groups. (G) Correlational analysis of the qPCR and RNA-seq results.

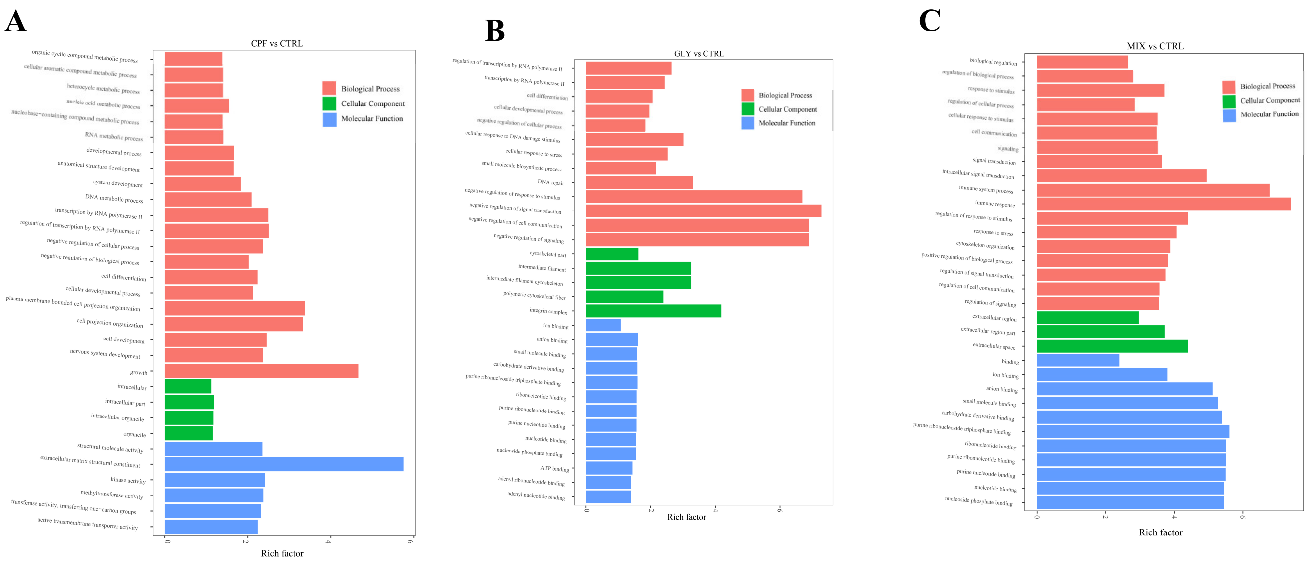

Figure S2. GO analysis of DEGs in CPF, GLY, and MIX treatment groups. (A-C) The DEGs in the CPF, GLY, and MIX group were classified by GO (The GO categories' functions were divided into biological processes, cellular components, and molecular functions).
